# Adalimumab‐responsive Monogenic Inflammatory Bowel Disease With Pseudopolyposis Characteristic of *TGFBR2* Variant in Loeys‐Dietz Syndrome

**DOI:** 10.1002/deo2.70266

**Published:** 2025-12-19

**Authors:** Tomomitsu Sado, Satoshi Ukai, Shingo Kurasawa, Yosuke Kono, Norio Hasuda, Mai Iwaya, Takaya Nakane, Tomomi Yamaguchi, Tomoki Kosho, Yoshiko Nakayama

**Affiliations:** ^1^ Department of Pediatrics Shinshu University School of Medicine Nagano Japan; ^2^ Department of Pediatrics Faculty of Medicine University of Yamanashi Yamanashi Japan; ^3^ Department of Surgery II Faculty of Medicine University of Yamanashi Yamanashi Japan; ^4^ Department of Pathology Shinshu University School of Medicine Nagano Japan; ^5^ Center For Medical Genetics University of Yamanashi Yamanashi Japan; ^6^ Department of Medical Genetics Shinshu University School of Medicine Nagano Japan; ^7^ Center For Medical Genetics Shinshu University Hospital Nagano Japan; ^8^ Division of Clinical Sequencing Shinshu University School of Medicine Nagano Japan; ^9^ Research Center for Supports to Advanced Science Shinshu University Nagano Japan; ^10^ BioBank Shinshu Shinshu University Hospital Nagano Japan

**Keywords:** adalimumab, colonic polyps, inflammatory bowel disease, Loeys‐Dietz syndrome, TGFBR2

## Abstract

Loeys‐Dietz syndrome (LDS) is an autosomal dominant connective tissue disorder caused by pathogenic variants in *TGFBR1* or *TGFBR2*. It is characterized by vascular fragility, skeletal abnormalities, and predisposition to allergic and inflammatory conditions, including monogenic inflammatory bowel disease (IBD). We report a pediatric case of IBD with a *TGFBR2* variant, presenting active colonic inflammation and pseudopolyposis, treated with adalimumab.

At age 7, the patient presented with abdominal pain and bloody stools. Upon referral, colonoscopy demonstrated mucosal fragility, prominent pseudopolyposis, and an anal fissure, accompanied by a skin tag was identified. Genetic analysis revealed a heterozygous *TGFBR2* c.1583G>A (p.Arg528His) variant and heterozygous *MEFV* E148Q and P369S‐R408Q variants. Colchicine treatment for suspected familial Mediterranean fever‐associated enteritis had a limited effect. Adalimumab treatment was initiated, leading to endoscopic improvement, with resolution of anemia and inflammatory markers in the blood test. This report presents the clinical course and endoscopic features potentially specific to the *TGFBR2* c.1583G>A variant, with reference to previously published cases.

## 1 Introduction

Loeys‐Dietz syndrome (LDS) is a systemic connective tissue disorder caused by pathogenic variants in *TGFBR1* or *TGFBR2*, characterized by arterial tortuosity, aneurysms, and skeletal and craniofacial abnormalities [1]. Its association with allergic and inflammatory diseases, including inflammatory bowel disease (IBD), has gained increasing attention [2]. Despite this, clinical reports remain scarce, particularly those detailing endoscopic and histopathologic features or treatment outcomes. This report describes a rare case of pediatric‐onset monogenic IBD in the setting of LDS, wherein initiation of adalimumab treatment was associated with marked improvement in endoscopic findings.

## 2 Case

**FIGURE 1:**
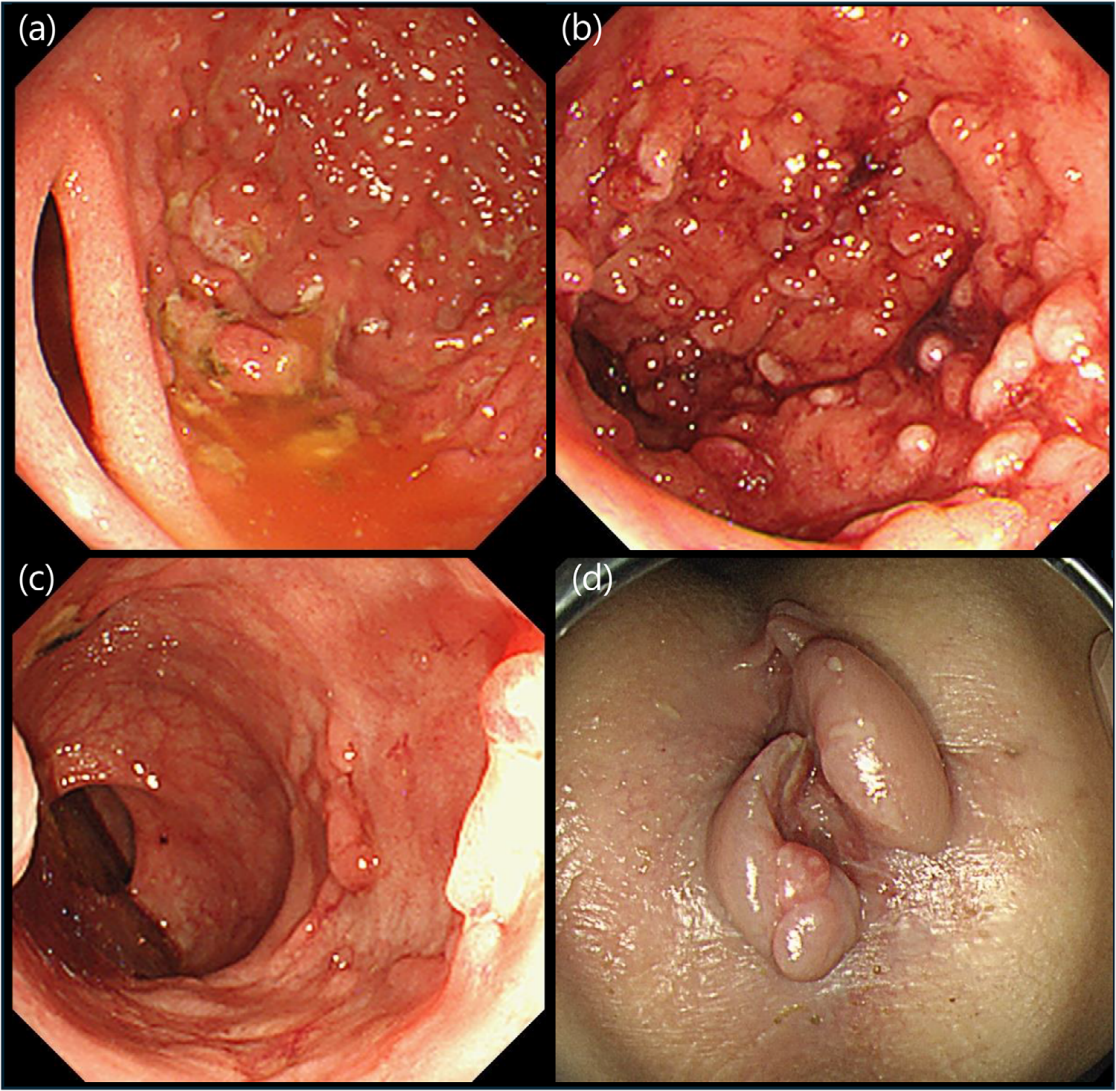
Colonoscopy images at presentation. Panel (a) shows the ascending colon, (b) the transverse colon, (c) the rectum, and (d) the anus during endoscopic examination. Continuous, circumferential, diffuse erythema was observed from the rectum to the ascending colon, accompanied by pseudopolyposis. Ulcer scar was noted in the rectum. In the anal region, an anal fissure accompanied by a skin tag was observed.

**FIGURE 2:**
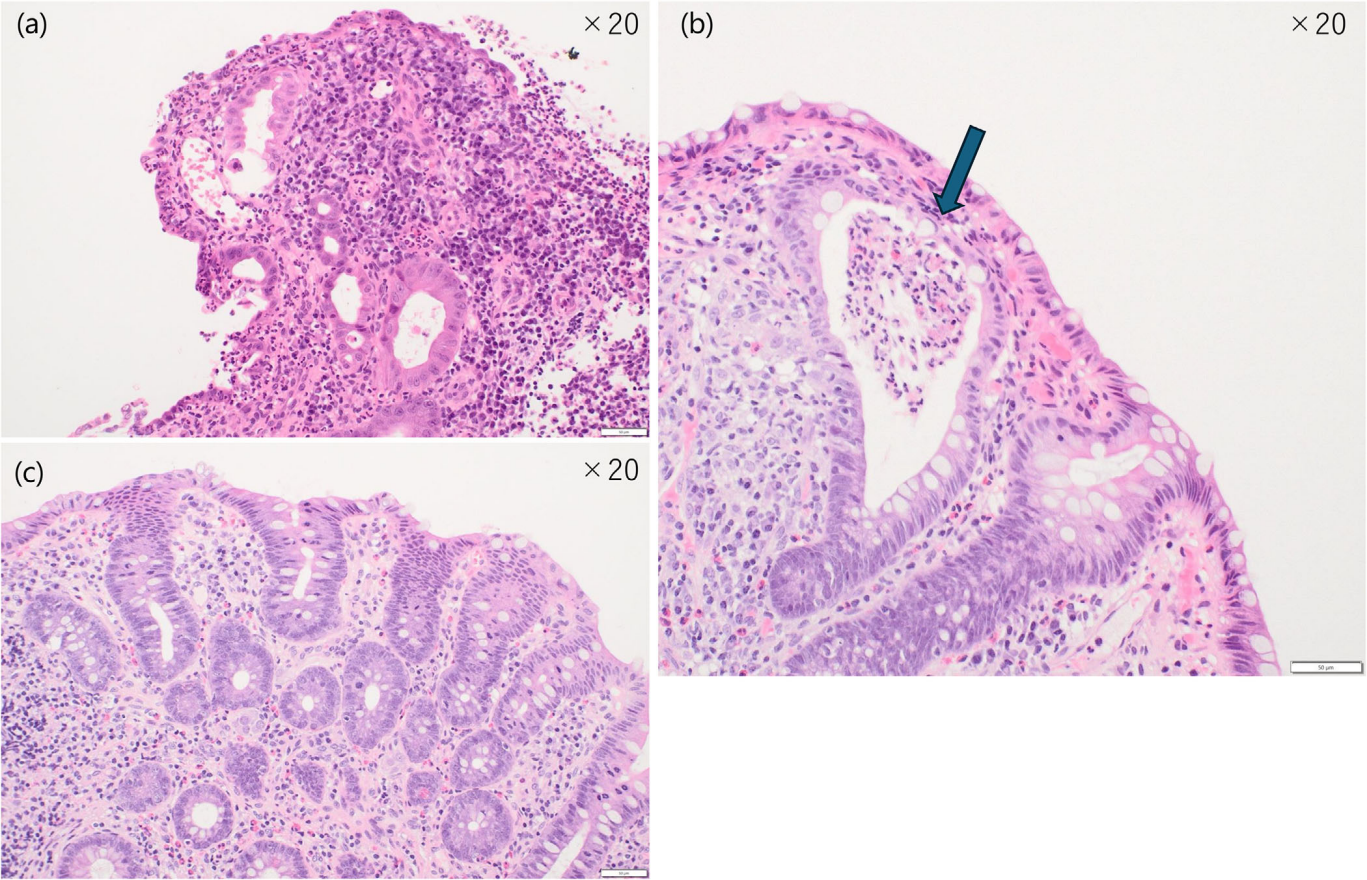
Hematoxylin and eosin (H&E) stained sections of the patient's colonic biopsy. Panel (a) shows the colonic mucosa at the initial presentation, obtained from a pseudopolyposis lesion in the cecum, demonstrating marked neutrophilic infiltration of the lamina propria. Panel (b) shows a crypt abscess in the rectum, indicated by an arrow. Panel (c) shows the findings three months after initiation of adalimumab treatment, demonstrating a marked reduction in inflammatory cell infiltration in the cecum.

**FIGURE 3:**
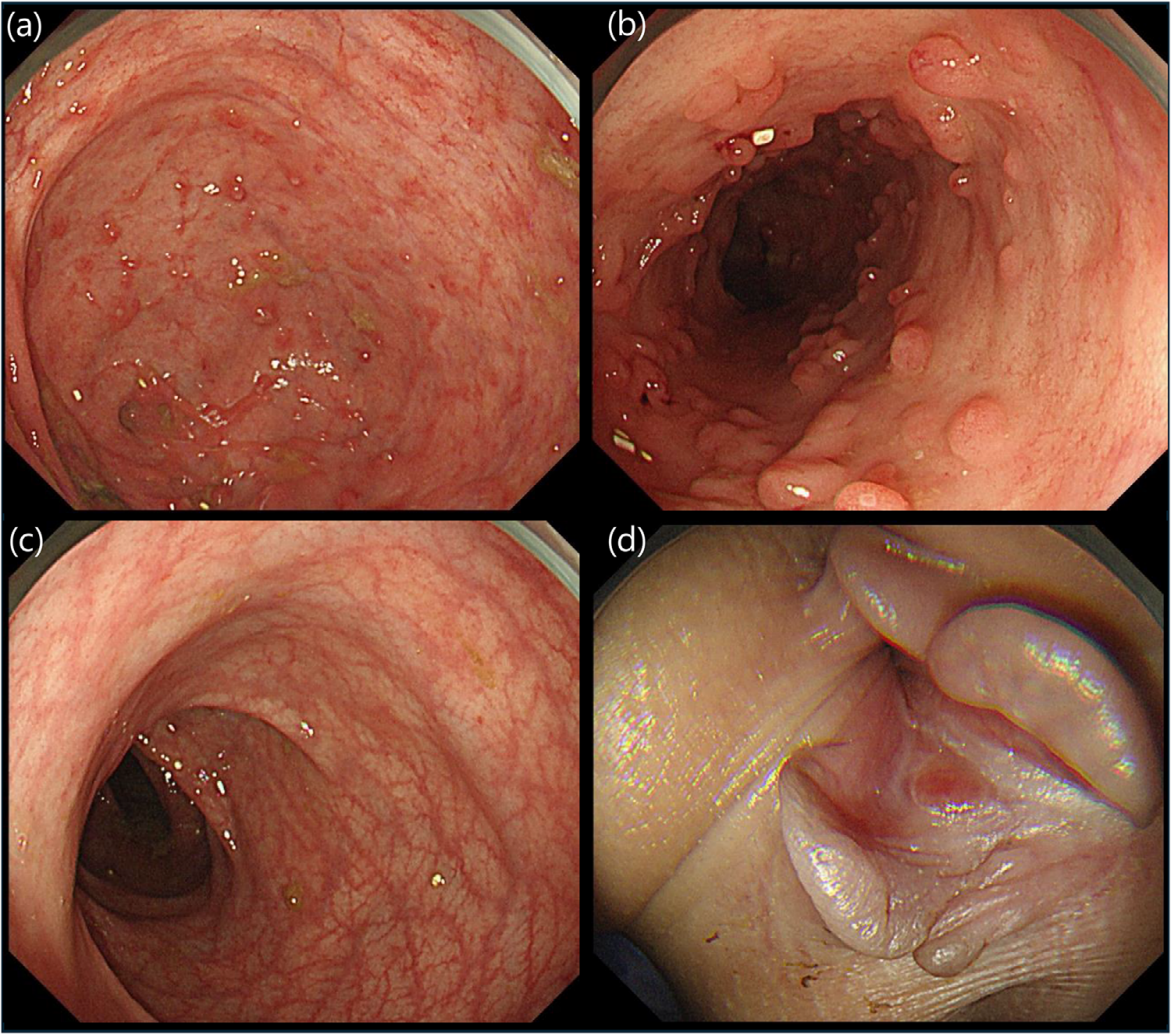
Colonoscopy images three months after adalimumab treatment. (a) shows the ascending colon, (b) the transverse colon, (c) the rectum, and (d) the anus on endoscopic examination. The pseudopolyposis showed a tendency to regress. The anal lesion demonstrated a gradual reduction in size.

An 8‐year‐old boy with a heterozygous *TGFBR2* (NM_003242.6):c.1583G>A (p.Arg528His) variant, genetically diagnosed as LDS, was referred to our department due to bloody stool, abdominal pain, and atypical endoscopic findings for IBD. His past medical history included congenital clubfoot, bilateral camptodactyly, multiple food allergies (peanuts, buckwheat, wheat, and milk), and a history of David procedure for aortic valve disease at age 8. He had been under treatment with losartan 15 mg/day. Symptoms of abdominal pain and bloody mucous stools started at age 7. At the initial presentation, his height was 126.4 cm (–1.8 SD), and his weight was 21.9 kg (–2.0 SD). His body temperature was 36.5°C, blood pressure 90/54 mmHg, pulse rate 78 beats/min, and oxygen saturation 98% on room air. Physical examination revealed conjunctival pallor and a perianal skin tag. Colonoscopy at that time showed diffuse mucosal redness, edema, and ulcers throughout the colon, leading to a diagnosis of pancolitis‐type ulcerative colitis (UC). Histopathology revealed infiltration of chronic inflammatory cells, along with neutrophils and eosinophils. Remission was initially achieved with oral 5‐aminosalicylic acid (5‐ASA) and prednisolone (1 mg/kg/day), but abdominal pain and bloody stools recurred upon steroid tapering. Upon referral, cessation of milk intake resulted in the resolution of hematochezia. Colonoscopy demonstrated mucosal redness and fragility, prominent pseudopolyposis from the sigmoid to ascending colon, and an ulcer scar was observed in the rectum (Figure 1a–c). An anal fissure accompanied by a skin tag was identified (Figure 1d). Histologically, pseudopolyposis showed crypt architectural distortion with neutrophilic infiltration of the lamina propria (Figure 2a). Cryptitis and crypt abscesses were observed (Figure 2b). Capsule endoscopy revealed no small bowel involvement. In addition to a known pathogenic *TGFBR2* c.1583G>A variant, heterozygous *MEFV* E148Q and P369S‐R408Q variants, and a heterozygous *NUDT15* Arg/Cys were also identified. Given the suspicion of familial Mediterranean fever (FMF)‐associated enteritis due to *MEFV* variants, oral colchicine (0.03 mg/kg/day) was initiated. Although a slight reduction in inflammatory cell infiltration was observed histologically, endoscopic findings showed no significant improvement. Laboratory evaluation revealed hypoalbuminemia (albumin, 3.2 g/dL), anemia (hemoglobin, 10.1 g/dL), and elevated inflammatory markers, including C‐reactive protein (CRP, 0.66 mg/dL) and erythrocyte sedimentation rate (ESR, 20 mm/h). Serum concentrations of IL‐1β, IL‐10, and IL‐12p70 were assessed using a commercially available cytometric bead array–based human inflammatory cytokine assay (BD Biosciences, San Jose, CA, USA), and all values were below the assay's limit of detection. Given the insufficient response to colchicine, adalimumab was administered at a standard induction and maintenance dose for pediatric UC (80 mg at week 0, 40 mg at weeks 1 and 2, then 40 mg every 2 weeks from week 4). After 3 months, laboratory findings improved, with albumin levels having normalized to 4.0 g/dL, hemoglobin to 12.2 g/dL, CRP declining to 0.06 mg/dL, and ESR to 5 mm/h. Follow‐up endoscopy showed decreased pseudopolyposis, improved mucosal vascular pattern, and reduced bleeding tendency (Figure 3a–3c). The anal lesion demonstrated a gradual reduction in size (Figure 3d). In histological examination, following adalimumab treatment, inflammatory cell infiltration, including neutrophils, was markedly reduced (Figure 2c). For one year following the initiation of adalimumab treatment, the patient has shown no clinical relapse or deterioration in laboratory parameters.

## 3 Discussion

Transforming Growth Factor‐β (TGF‐β) is a multifunctional cytokine essential for regulating cell differentiation, proliferation, immunity, and extracellular matrix maintenance. TGF‐β binds first to the type II receptor (TGFBR2), which activates the type I receptor (TGFBR1). Activated TGFBR1 phosphorylates SMAD2/3, allowing them to form a complex with SMAD4 and translocate to the nucleus to regulate gene transcription. Disruption of this pathway underlies the connective tissue abnormalities and immune dysregulation characteristic of LDS. LDS due to *TGFBR1* variants is classified as type I, and due to *TGFBR2* variants as type II, the latter generally exhibiting a more aggressive aortic phenotype [3].

In a cohort of 93 patients with genetically confirmed LDS, 4 individuals (4.3%) developed IBD, a prevalence approximately five times higher than that of the general adult population [4]. In the same cohort, 1 of 30 patients with LDS type I (3.3%) developed IBD, compared with 3 of 63 patients with type II (4.7%).

In the present case, a heterozygous *TGFBR2* c.1583G>A variant, resulting in a p.Arg528His substitution, was identified. Among previously documented cases of IBD associated with *TGFBR1* or *TGFBR2* variants (Table 1), two cases were documented to possess the *TGFBR2* c.1583G>A variant. Of the two cases, one demonstrated diffuse colonic inflammation with pseudopolyposis [5], while the other showed endoscopic findings limited to inflammatory polyps [4]. In both reported cases, adalimumab administered during the active phase failed to induce a favorable clinical response. To the best of our knowledge, although there have been reports of patients with *TGFBR2* variants developing IBD, pseudopolyps have not been described in cases with variants other than c.1583G>A. The *TGFBR2* c.1583G>A variant is registered in ClinVar as pathogenic for LDS type II, but there is no registration indicating its potential association with susceptibility to IBD. This is the first report to describe distinctive endoscopic findings observed in both the active phase and the treatment‐responsive phase of IBD associated with the *TGFBR2* c.1583G>A variant.

**TABLE 1:** Eight monogenic IBD cases with *TGFBR1* or *TGFBR2* variants have been reported. Two cases of the p.Arg528His phenotype were reported. In this phenotype, polypoid changes were observed in all cases.

No.	Article	Age onset	Sex (F, M)	Gene	variant	Colonoscopic findings	Course of treatment
1	2	1y	F	*TGFBR2* (NM_003242.6)	c.1582C>T,p.Arg528Cys	Fragile, edematous, and diffusely ulcerated	Steroid‐dependent, infliximab, and losartane were ineffective
2	2	2y	M	*TGFBR1* (NM_004612.4)	p.Gly217Arg	Diffusely congested, edematous, and ulcerated	Steroid dependent, but became steroid resistant, ineffective to adalimumab treatment
3	4	3m	F	*TGFBR2*	c.1583G>A,p.Arg528His	Active inflammation with inflammatory polyps	Symptoms improved with 6‐MP, then relapsed, unresponsive to infliximab and adalimumab, but improved with sirolimus
4	4	6y	M	*TGFBR1*	c.1058G>T,p.Gly353Val	Normal endoscopic findings	Unresponsive to 6‐MP, infliximab, adalimumab, and methotrexate, then improved with sirolimus
5	^4^	15y	F	*TGFBR2*	c.1579G>A,p.Ala527Thr	Active ileitis	Not specified
6	4	27y	F	*TGFBR2*	c.1379G>A,p.Arg460His	Not specified	Steroid dependent
7	5	1y	M	*TGFBR2*	c.1583G>A,p.Arg528His	Diffusely ulcerated with multiple pseudopolyposis	Steroid dependent, unresponsive to adalimumab, improved with ustekinumab
8	^a^	7y	M	*TGFBR2 MEFV* (NM_000243.2)	c.1583G>A,p.Arg528His E148Q and P369S‐R408Q	Redness and fragility with pseudopolyposis, rectal ulcer scars, and a submucosal tumor‐like lesion	Steroid‐dependent with limited efficacy of 5‐ASA, unresponsive to colchicine, improved with adalimumab^b^

Abbreviations: 6‐MP, 6‐mercaptopurine; F, female; M, male; m, months; y, years.

^a^ This case.

^b^ Colchicine was continued during adalimumab treatment.

Our patient also harbored MEFV E148Q and P369S–R408Q variants, which initially raised the possibility of FMF‐associated enterocolitis. A case of FMF‐associated enterocolitis presenting with pseudopolyposis has been reported; however, the pathogenic variant (*MEFV* 910G>A) differed from that in the present case, and marked endoscopic improvement was observed following colchicine [6]. The variants *MEFV* E148Q and P369S‐R408Q are widely recognized as low‐penetrance alleles frequently found in healthy individuals [7, 8]. Given the generally good responsiveness of FMF‐associated enterocolitis to colchicine, the presence of these variants alone does not strongly support FMF‐associated enterocolitis as the primary etiology in this case. Colchicine‐refractory cases of FMF‐associated enterocolitis are frequently associated with the presence of an *MEFV* M694V variant; however, most cases without the M694V variant demonstrate a favorable response to colchicine treatment [9].

We selected adalimumab for treatment because anti–TNF‐α therapy is widely used as a first‐line biologic treatment in monogenic or genetically determined enterocolitis when sustained mucosal inflammation or steroid dependence is present [10]. In our patient, colchicine was continued during adalimumab therapy, and a slight histological reduction of inflammatory cell infiltration was observed before biologic initiation. Although *MEFV*‐related inflammation was unlikely to be the major driver of the disease, the combined use of colchicine may have contributed to partial control of the inflammatory milieu, potentially facilitating a more favorable therapeutic response to adalimumab. Additionally, in previously reported patients carrying the same *TGFBR2* c.1583G>A variant, gastrointestinal symptoms developed in early infancy (3–4 months and 1 year) (Table 1), whereas our patient presented at 7 years of age. Differences in the timing of disease onset might partly underlie variations in treatment response to adalimumab.

This case demonstrates a favorable therapeutic response to adalimumab in monogenic IBD associated with *TGFBR2* c.1583G>A and delineates serial endoscopic changes from the active to the treatment‐responsive phase. Among the reported cases of IBD associated with LDS, the most frequently identified variant was *TGFBR2* c.1583G>A, detected in three of eight cases (37.5%). This finding suggests that when the p.Arg528His variant is identified during genetic testing for LDS, patients should be carefully monitored for the potential future development of IBD. Although adalimumab has been administered to four previously reported cases without a favorable response, our case represents the first to demonstrate clinical and endoscopic improvement, highlighting the need for accumulating detailed case reports to clarify variant‐specific endoscopic patterns and guide therapeutic strategies for rare disorders.

## Author Contributions

**Tomomitsu Sado**: conceptualized and designed the study, conducted the investigation, performed methodology, validation, visualization, and formal analysis, and drafted the original manuscript. **Satoshi Ukai**, **Shingo Kurasawa**, **Yosuke Kono**, **and Norio Hasuda**: contributed to formal analysis. **Mai Iwaya**: contributed to formal analysis, visualization, and data curation. **Takaya Nakane**: contributed to formal analysis, writing – review & editing, and data curation. **Tomomi Yamaguchi**: contributed to formal analysis, writing – review & editing, and data curation. **Tomoki Kosho**: contributed to formal analysis, writing – review & editing, supervision, and data curation. **Yoshiko Nakayama**: contributed to supervision, formal analysis, and writing – review & editing. All authors read and approved the final version of the manuscript and agree to be accountable for all aspects of the work.

## Funding

The authors received no specific funding for this work.

## Conflicts of Interest

Tomomi Yamaguchi and Tomoki Kosho are members of an endowed chair named “Division of Clinical Sequencing, Shinshu University School of Medicine” sponsored by BML, Inc. The other authors declare no conflicts of interest.

## Ethics Statement

The patient in this case report was treated within the standard scope of care under Japan's national health insurance. Informed consent was obtained from the patient for the medical treatment and procedures described. In publication, all accompanying images and videos have been anonymized to ensure that the individual cannot be identified.
